# Exploring Factors Affecting Urban Park Use from a Geospatial Perspective: A Big Data Study in Fuzhou, China

**DOI:** 10.3390/ijerph20054237

**Published:** 2023-02-27

**Authors:** Liguo Zeng, Chunqing Liu

**Affiliations:** 1 College of Landscape Architecture and Art, Jiangxi Agricultural University, Nanchang 330045, China; 2 College of Resources and Environmental Sciences, Quanzhou Normal University, Quanzhou 362000, China

**Keywords:** urban park use, park-surrounding facilities and services, geographic detector, multiscale geographically weighted regression, real-time Tencent user density, Fuzhou

## Abstract

Promoting research on urban park use is important for developing the ecological and environmental health benefits of parks. This study proposes uniquely integrated methods combined with big data to measure urban park use. It combines comprehensive geographic detectors and multiscale geographically weighted regression from a geospatial perspective to quantify the individual and interactive effects of the parks’ characteristics, accessibility, and surrounding environment features on weekday and weekend park use. The study also explores the degree of influence of spatial changes. The results indicate that the park-surrounding facilities and services factor contributed most to use, while its interaction effect with park service capacity had the greatest impact on park use. The interaction effects showed binary or nonlinear enhancement. This suggests that park use should be promoted within multiple dimensions. Many influencing factors had significant changes in the geographic space, suggesting that city-level park zoning construction should be adopted. Finally, park use was found to be affected by users’ subjective preference on weekends and convenience factors on weekdays. These findings provide a theoretical basis for the influencing mechanisms of urban park use, which can help urban planners and policymakers formulate more specific policies to successfully manage and plan urban parks.

## 1. Introduction

As an important part of the urban ecosystem, urban parks improve the urban built environment by purifying air [1,2], controlling noise pollution [3], mitigating the heat island effect [4], and preserving biodiversity [5,6]. In addition, urban parks provide ecological and cultural services such as aesthetic sightseeing, recreation and entertainment, education and culture, and sports and social communication [7,8] for urban residents, thus improving their quality of life. Recent studies have also focused on the concept of parks as places where residents can stay safely in the post-pandemic period after 2020 [9,10,11]. However, the environmental health benefits of urban parks that increase people’s well-being can only be realized when people use the park. Therefore, urban park use and the factors that affect urban park visits have attracted increasing attention from both policymakers and urban planners.

Urban park use refers to the actual visitation of urban parks by residents in their daily lives [12]. A series of recent studies have proven that park characteristics, accessibility, and surrounding environment features constitute the three factors that affect park visits [12,13,14]. Park characteristics, such as park size (P-SI), park type (P-TY), landscape shape index (LSI), water size (W-SI), park facilities (P-FA), and park service capability (PSC), have a positive relationship with park use [15,16,17]. Accessibility, whose positive role in the promotion of urban park use is often mentioned in urbanized society [16,18], determines whether visitors can easily reach urban parks [19]. Related accessibility factors include the distance to the city center [17] (DTC) and bus station density [20] (BSD). A specific evaluation indicator is the real-time accessibility of areas when walking (about 15 min) and driving (about 30 min), which is evaluated using open data [21]. Moreover, surrounding environment features such as population density, which can be divided into resident and working population density (RPD and WPD, respectively), and park-surrounding services and facilities (SPOI) all contribute to increased park visitation [17,20,22,23].

Regarding data collection for urban park use, traditional methods, such as social surveys [24] or systems for observing play and recreation in communities (SOPARC) [25], have investigated urban park users’ characteristics, activities, and behaviors to generate detailed information through questionnaires or observation. However, these require significant time, labor, and funding [20]. Moreover, the validity and reliability of questionnaire surveys are at risk of implicit bias [15], while SOPARC cannot provide much information about park management at the city level [26]. The recent use of geospatial data, such as geolocated social media or mobile phone signaling data, has created new opportunities to measure park use frequency in a more convenient and cost-efficient manner [27,28]. For example, many social media platforms (e.g., Flickr, Instagram, Twitter, and Weibo) support check-in options and text broadcasts that require users to actively upload information that can be used to track visitors’ real-time locations and feelings about specific parks [29]. As geolocated social media data are highly contingent on tagging by users, it is a passive way to obtain location data; however, it cannot provide urban park use data at daily or hourly intervals [17]. Instead, mobile phones can be programmed to automatically send users’ location information to base stations every half hour to update users’ status [13], thus helping researchers to comprehensively and accurately deduce the spatiotemporal distribution of park visitors. However, as the spatial resolution of mobile signaling data is about 100–300 m [30], it is only suitable for collecting data on visitors in large urban parks of more than 10 ha. Therefore, it is invalid for use in small- and medium-sized parks that are widely distributed and frequently used in cities [31].

Compared with traditional methods for data collection and geospatial data mentioned above, real-time Tencent user density (RTUD) is another big data source that can be used in an equitable and unbiased manner to provide higher precision and real-time population dynamic data. Recent studies have employed RTUD to map urban population distribution at the building scale in the study of land use [32], urban structure [33], and population migration [30]. However, few studies have applied these data to the measurement of park use at the city level [20] even though they have been shown to do a far better job than other data in revealing how many people use parks of any size (except mini ones) [34].

Regarding research perspectives and methodologies, previous research has mainly used traditional regression models [12,20], such as multiple linear regression, to explore the factors affecting park use but has often ignored the multicollinearity among influencing factors and the spatial relationship between driving factors [35]. This has led these studies to miss important details and insufficiently reflect the actual phenomena that reveal the correlations between park use and its influencing factors [20,23,34]. Moreover, recent studies have shown that the relationship between park use and its driving factors is not linear but rather shows significant spatial heterogeneity [14,15,17,20]. Geographical detectors and multiscale geographically weighted regression (MGWR) are two new spatial statistical methods that can be integrated to quantify spatial heterogeneity and investigate the spatial pattern of determinants of geospatial phenomena. These have been applied in several fields, including ecosystem services [36], housing prices [37], and PM2.5 concentration coverage [38]; however, there have been few attempts to use these methods to examine the relationship between urban park use and its influencing factors. Therefore, using these integrated methods to analyze park use according to various influencing factors from a geospatial perspective is essential for formulating targeted urban park planning and optimizing park management.

Considering the limitations of the previous research mentioned above, the specific objectives of this study are (i) to measure park use using RTUD data and characterize park use in the main urban area of Fuzhou; (ii) to determine influencing factors and obtain data from multiple sources; (iii) to analyze the comprehensive response of park use to various drivers and their spatial differences by integrating geographical detectors and MGWR; and (iv) to recommend measures for improving park use in cities of similar size to Fuzhou. By utilizing RTUD data as a new way of measuring urban park use, this study proposes an integrated method from a geospatial perspective to reach a deeper understanding of park use and provides significant suggestions for urban park management and planning.

## 2. Methodology

### 2.1. Study Area

Fuzhou, the capital of Fujian Province, is a coastal city in southeast China. According to the Fuzhou Statistical Bureau Survey, as of 2021, the city’s administration area covers 11,968 km^2^ and has a total population of 8.42 million [39]. To ensure data availability, this study selected Fuzhou’s main urban area as the study area. This includes four districts that cover a total area of 398 km^2^: Gulou, some parts of Jin’an, Taijiang, and Cangshan. This study focused on 98 parks ranging in size and type across the study area to represent a diverse sample.

**Figure 1:**
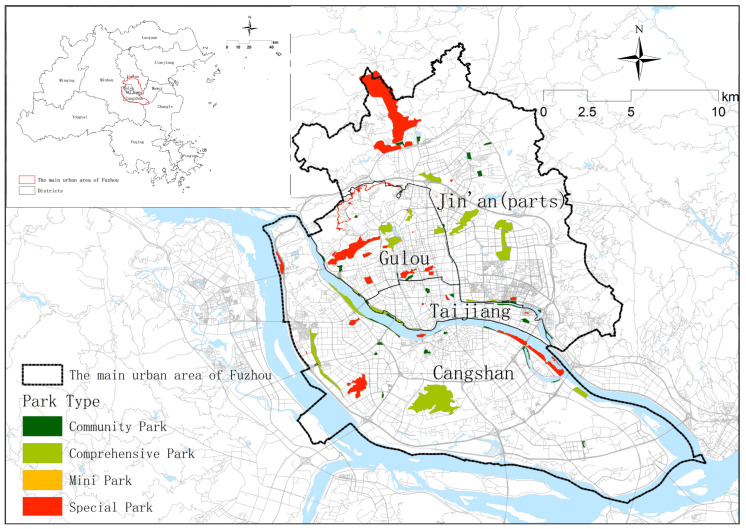
Study area: The main urban area of Fuzhou.

After obtaining the boundary of Fuzhou’s main urban area from the WGS1984 coordinate system using the professional map software Shuijing MicroMap, this study imported the vector file into the ArcGIS 10.6 platform. The urban parks in Fuzhou’s main urban area were then search for using the polygon search interface on an open platform application programming interface called AMAP (http://ditu.amap.com, accessed on 16 September 2021). This is one of the most popular route-planning service providers in China, with up-to-date remote sensing data and fine granularity. The park names, coordinates, and central coordinates were obtained. The parks’ boundary information was determined using the POIinfo interface. Overall, 98 urban parks’ polygon shapefiles were located. According to the Standard for Classification of Urban Green Space [40], the parks were then classified into comprehensive parks (n = 18), community parks (n = 36), special parks (n = 29), and mini-parks (n = 15) (Figure 1). Comprehensive parks are rich in content and suitable for various activities, special parks have a specific content or form, community parks provide places for residents to do activities nearby, and mini-parks allow visitors to briefly rest. These different types of parks are also different in function, scale, and management, representing different samples of parks in the main urban area of Fuzhou (Figure 1).

### 2.2. RTUD (Real-Time Tencent User Density) Data and Processing

RTUD data feature a spatial resolution of 25 m and can meet the accuracy standard for all park sizes, except mini-parks smaller than 1600 m^2^ [34]. RTUD data were considered the most suitable for tracing park visitation. When people use Tencent apps on their mobile devices, including QQ, WeChat, and Tencent Map, their location information is recorded in shapefile format on Tencent’s web GIS platform. Tencent’s location big data interface (https://heat.qq.com/bigdata/index.html, accessed on November 2021) can be used to apply for this RTUD data. The number of users in each 25 m resolution pixel unit was then counted, thus forming park visitation data. As WeChat has 1.26 billion monthly active accounts, representing the majority of China’s 1.44 billion population [41], RTUD data are the most reliable source of geographic information about populations in Chinese cities [30].

To guarantee the effectiveness and representativeness of the data, this study gathered hourly RTUD data for the 98 parks in the study area on Wednesdays (representing weekday visits) and Saturdays (representing weekend visits) for three consecutive weeks in November 2021. The measured days were sunny and breezy with temperatures between 15 and 25 °C, ideal weather for outdoor activities. There were also no park festivals that resulted in abnormal crowds. To evaluate the accuracy of the RTUD data, ten parks were selected (three larger than 10 ha, five between 1 and 10 ha, and two smaller than 1 ha) to conduct a field survey. The authors manually counted the number of park visitors per hour in their respective zones from 06:00 to 22:00. The results showed an 83.55% consistency rate with the RTUD data. Considering that some park visitors might not use Tencent apps on their mobile devices, this formed an acceptable margin of error; thus, this study concluded that the RTUD data could mostly reflect real-time park visitation.

Further, to minimize the interference of random factors that may have altered the values of park visitation, this study used the mean hourly value traced during peak visitation hours. After reviewing the variations between each park’s hourly RTUD data, this study found that the peak point of traced visits for all parks was 15:00–17:00 on Wednesdays (Figure S1) and 16:00–18:00 on Saturdays (Figure S2). This difference may be because people try to avoid the 18:00 rush hour on weekdays. Therefore, this study calculated the average number of hourly visits and divided it by the area of each park to obtain active user density from 17:00 to 19:00 on three consecutive Wednesdays and between 16:00 and 18:00 on three consecutive Saturdays.

Next, this study adopted a reproducible two-factor nested experimental design [42] to investigate the impact of each factor on the measurement results of park use density (PUD). One factor was the difference between weekdays (represented by Wednesday data, mentioned above) and weekends (represented by Saturday data, mentioned above); the other factor was the difference between parks. This calculation process was carried out in SPSS software, and an analysis of variance showed that, at the 0.05 significance level (*p*-values in Table S1 are all 0), both F_parks_ and F_weekdays and weekends_ were larger than their respective F_crit_ (Table S1). Therefore, this study concluded that both the differences between parks and between weekdays and weekends significantly impacted the measurement results of PUD [43]. Differences in geospatial location led to different user densities in different parks [44], while differences in the composition and recreational pattern of park visitation led to differences between weekdays and weekends [45]. Accordingly, this study performed two series of regression models to study park use on weekdays and weekends, and PUD was adopted as a dependent variable (Table 1) for further analysis.

### 2.3. Selection of Influencing Factors and Data Sources

Based on a literature review [12,14,15], this study introduced three groups of independent variables that could potentially affect urban park use: park characteristics, accessibility, and surrounding environment features (Table 1).

Park characteristics pertained to the park itself and included P-SI, P-TY, LSI, W-SI, P-FA, and PSC. P-SI was calculated based on the polygon shapefiles formed in Section 2.1, while P-TY is also explained in that section. LSI was used to reflect the fragmentation degree of the park boundaries, and was calculated as follows:

$$\mathrm{LSI}=2\frac{\sqrt{\pi \times Si}}{Ci}$$

where *Si* represents the area of the urban park *I* in hectares and *Ci* signifies the circumference of the park *I* in meters. W-SI refers to the area of water within the park. P-FA data, including playgrounds, themed plazas, lounge corridors, restaurants, shops, toilets, parking lots, and others, were taken from field research. The evaluation of PSC came from the overall rating data on the parks’ scenery, hygiene, safety, and other services from Dianping (www.dianping.com, accessed on 7 December 2021) users who visited the park; Dianping is one of the leading social media platforms for sharing local lifestyle information in China.

**Figure 2:**
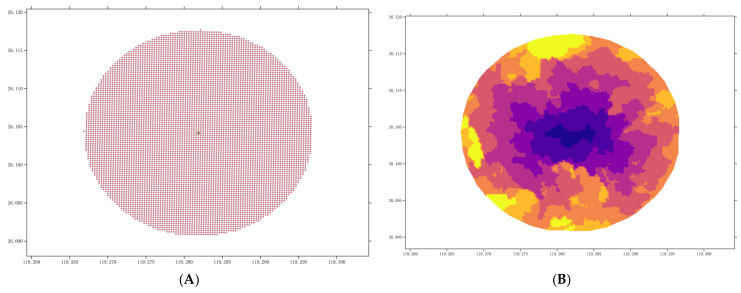

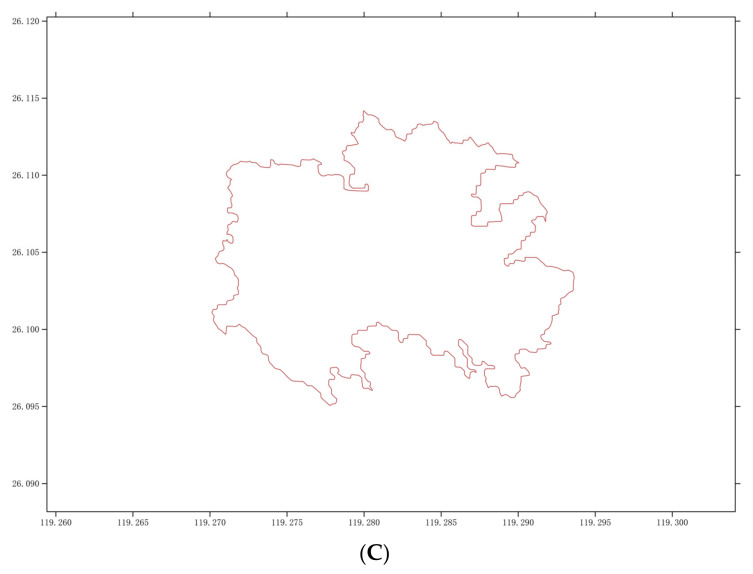
Examples of control point interpolation data. (**A**) Control point data. (**B**) Interpolation raster data. (**C**) Track contour line data.

To avoid the disadvantages of subjective assignments in previous park accessibility measures, this study used AMAP’s real-time path planning tool to calculate area accessibility from 15 min of walking (W-15) and 30 min of driving (D-30) in an isochronous circle during non-peak hours on weekdays [21]. Figure 2 shows that starting from the parks’ parking lot, control points are set in the north–south and east–west directions (Figure 2A). The driving distance from each control point to the start point was obtained through AMAP’s path planning interface. This study used the Matplotlib library and SciPy for cubic interpolation to obtain a grid file describing the timed distance to the park (Figure 2B) and then calculated the area within the boundary according to the contour line of the 30 min grid file (Figure 2C). Simultaneously, W-15 started from the entrance of the park. For parks with multiple entrances or parking lots, the isochronous circles of each starting point were combined. Moreover, DTC and BSD were also used to represent park accessibility [46]. This study set Fuzhou Wuyi Square as the city center and calculated its distance from the central point of the park. Using the Standard for Planning of Urban Green Space [47], this study measured the surrounding features within 1200 m for comprehensive parks and special parks, 500 m for community parks, and 300 m for mini-parks and delimited buffer zones based on the distance. In total, data for 6580 bus stations that intersected each park’s buffer zone were collected from the Fuzhou public transportation website (http://fuzhou.gongjiao.com/, accessed on 12 November 2021); the data were divided by the buffer zone to obtain BSD.

The surrounding environment features were structured by the RPD and WPD within each park’s buffer zone alongside the services and facilities, which were correlated with park visitation [17,20,22,23]. As detailed population data are not always available through censuses, mobile phone signaling data were employed to identify the RPD and WPD. Specifically, this study considered the spatial locations of the sample in which mobile phone signals were silent from 0:00 to 5:00 as the places where the sample resided and regarded the appearance of the sample from 10:00 to 16:00 on three consecutive days as the sample of WPD [21]. SPOI was represented by point of interest (POI) density; this study collected POI data using POI coordinates and attributes from AMAP in 2021, mainly in seven categories (catering services, shopping services, life services, education and culture services, medical care services, sports and leisure services, and accommodation ser-vices) that have positive effects on the use of parks. Similar to the process for determining BSD and RPD, this study screened all POIs located within a network distance of 1200, 500, or 300 m from the park boundaries using the network analysis and spatial join tools in ArcGIS 10.6 and then divided them by buffer zones to obtain POI density.

### 2.4. Geographical Detectors

Geographical detectors are effective spatial statistical methods that can detect spatial stratification heterogeneity to reflect variable similarity within the same region and variable differences in distinct regions, thus revealing the potential influencing factors [48]. They may comprise as many as four modules, though this study mainly focused on two: factor and interaction detectors.

The factor detector can detect the effect of influencing factors (Xn) on park use (Y) based on the q-statistic; the formula is as follows [48]:

$$q=1-\frac{\sum_{h=1}^{L}N_{h}\sigma_{h}^{2}}{{N\sigma }^{2}}=1-\frac{\mathrm{SSW}}{\mathrm{SST}}$$

$$\mathrm{SSW}=\sum_{h=1}^{L}N_{h}\sigma_{h}^{2}\;\mathrm{SST}={N\sigma }^{2}$$

where q represents the explanatory ability of influencing factors (Xn) on park use (Y); *h* = “1, 2, 3, …*L*” is the classification or stratification of variables; *N* and *N*_h_ are the number of units in layer *h* and the whole region, respectively; $\sigma_{h}^{2}$ and $\sigma^{2}$ denote the variance in units in *h* and the global variance in *y* over the whole region, respectively; *SSW* represents the sum of squares; and *SST* represents the total sum of squares. The greater the value of q, the stronger the effect of independent variable X on Y; q ranges from 0 to 1.

The interaction detector examines whether the factors (X_1,_ X_2_ … to X_s_) interactively affect urban park use. First, the q-statistic of Y is calculated for two factors, X_1_ and X_2_, and marked as q(X_1_) and q(X_2_); the q-statistic of their interaction (i.e., the new polygon distribution formed by the tangent of the two layers of the superimposed variables X_1_ and X_2_ (Figure S3)) is then calculated and marked as q(X_1_∩X_2_). Subsequently, q(X_1_), q(X_2_), and q(X_1_∩X_2_) are compared to obtain the interactive relationship results, which can be classified into five types (Table S2). These calculations are processed using the geographic detector software invented by Jinfeng Wang, which can be download for free.

Geographical exploration methods do not have a linear assumption and excel at the analysis of type quantity as the independent variable (such as P-TY in this study) and numerical quantity as the dependent variable (such as PUD) [49]. Therefore, for the numerical independent variables, this study used the natural breaks method in ArcGIS 10.6 to stratify the variables into seven layers (scores ranged from 1 to 7) and then used the geographical detectors for the statistical analysis. Meanwhile, in order to align with this value, we set the values for comprehensive parks (=7), special parks (−5), community parks (=−3), and mini-parks (=1).

### 2.5. MGWR

Geographically weighted regression (GWR) establishes local regression equations at each point in a spatial range and allows the relationships between independent and dependent variables to vary spatially. However, GWR assumes that each relationship varies at the same spatial scale and can be described by the same bandwidth parameter [50], which is often a misrepresentation of reality as the factors that affect a variable across space often do so at different spatial scales.

In response to this limitation of GWR, Fotheringham proposed MGWR [51]. Based on spatial heterogeneity, MGWR also considers spatial multiscale effects and allows for the estimation of local regression coefficients of dependent and independent variables on different spatial scales to produce a more realistic and useful spatial process model. The formula is as follows [51]:

$$y_{i}=\beta_{0}\left(u_{i},v_{i}\right)+\sum_{j=1}^{p}\beta_{\mathrm{bwj}}(u_{i},v_{i})x_{\mathrm{ij}}+\varepsilon_{i},i\epsilon \{1,2,\ldots ,n\}$$

where *y* is the dependent variable (PUD), $\left(u_{i},v_{i}\right)$ denotes the spatial location of the *i*-th sample, $\beta_{0}\left(u_{i},v_{i}\right)$ is the intercept, *p* is the number of independent variables (including park characteristics, accessibility, and surrounding environment features), $x_{\mathrm{ij}}$ represents the independent variables, $\beta_{\mathrm{bwj}}\left(u_{i},v_{i}\right)$ is the regression coefficient of the *i*-th sample for the *j*-th independent variable, and $\varepsilon_{i}$ is the error term. Moreover, bwj in $\beta_{\mathrm{bwj}}$ indicates the bandwidth used for calibration of the *j*-th conditional relationship, and each variable has its own specific bandwidth.

In this study, both the MGWR and GWR models used the Gaussian kernel function and the golden section method to search the bandwidth. All processing was conducted using MGWR 2.2 software (Jinfeng Wang, Beijing, China) [52].

## 3. Results and Analysis

### 3.1. Spatial Characteristics of Urban Park Use via RTUD Data

This study calculated the PUD of the 98 parks in Fuzhou’s main urban area for a weekday and a weekend group to reveal an average value of 26.23 people/ha·h with a standard deviation of 24.63 and an average value of 29.77 people/ha·h with a standard deviation of 26.16, respectively. The weekend group values were slightly higher than those of the weekday group.

**Figure 3:**
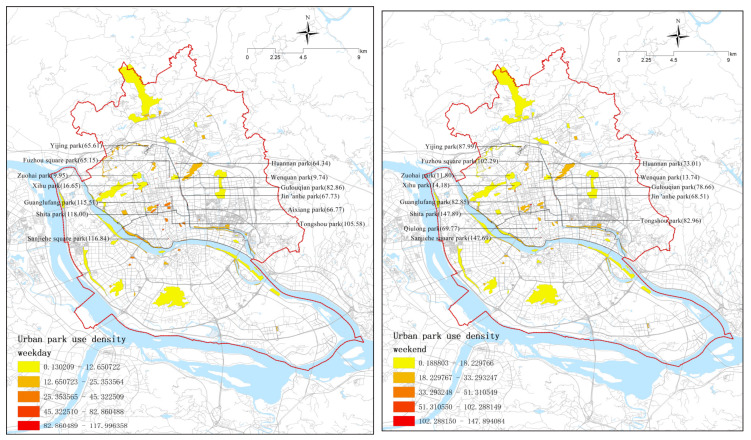
Spatial distribution of urban park use based on RTUD data.

Figure 3 shows that urban park use greatly varies by location and ranges from 4.6 to 147.89. It is obvious that, of the two groups, urban parks located closer to the city center have higher PUD. The top 10 parks with higher PUD are regarded as the most visited urban parks in Fuzhou. Irrespective of weekdays or weekends, these are small parks with good facilities and characteristics located in the city center. Most are based near historical sites, while some are located in popular riverside areas. Moreover, the PUD of large comprehensive parks, such as Xihu Park (16.65 on weekdays and 14.18 on weekends), Zuohai Park (9.95 and 11.88), and Wenquan Park (9.74 and 13.74), is much lower than that of smaller parks in the same location, which may be because the PUD index represents the visit intensity of urban parks rather than the total number of visits.

**Figure 4:**
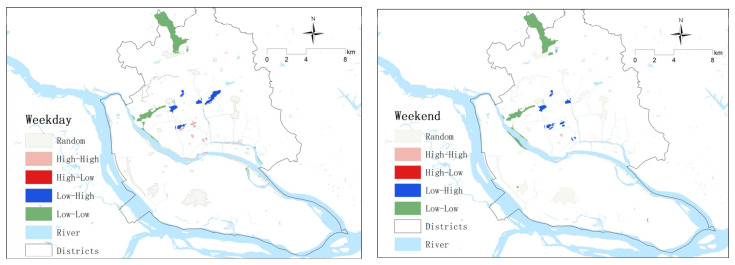
Local indicators of spatial association (LISAs) map of park use density (PUD) in Fuzhou.

In addition, this study also used local indicators of spatial association (LISAs) to identify the agglomeration of park use density in the two groups (Figure 4). “High-High” refers to a high-density cluster, indicating that a park with a high number of users is sur-rounded by similar parks. Additionally, “Low-Low” refers to a low-use density cluster. Further, “High-Low” and “Low-High” refer to parks surrounded by significant differences in use density. On weekdays, there are obvious clusters of high use density in the city center and obvious clusters of low use density in fringe areas. On weekends, the parks in the city center showed a significant difference in use density due to competition, with the clusters of high use density decreasing and the clusters of low use density remaining located in the marginal areas.

### 3.2. Quantitative Attribution of Park Use via Georaphical Detectors

#### 3.2.1. The Influence of Driving Factors on Urban Park Use

This study adopted the factor detector to quantify the influences of the 13 driving factors on the two groups’ urban park use. The results are shown in Table 2. At the 0.05 significance level (*p*-value < 0.05), LSI and BSD had no significant effect on park use in the two groups, which shows that the diversity of park boundaries and the convenience of public transportation did not significantly influence people’s choice of park. This is mainly because many parks in Fuzhou are built around scenic locations, which naturally have complex boundaries. The main urban area is not large, and the coverage of public transport stations is wide and balanced. Therefore, parks in Fuzhou showed little difference in LSI and BSD. W-SI and PSC were not significant in the weekday group but were significant in the weekend group, while W-15 was not significant in the weekend group but was significant in the weekday group. This may be because people usually have in-creased free time on weekends and therefore more flexibility in choosing parks. Thus, they were more inclined to choose parks based on their subjective preference, which may include Dianping or parks with a large water area. Conversely, on weekdays, people chose parks based on convenience factors, such as walking distance. The remaining eight independent variable factors all significantly influenced urban park use, although the extent of influence varies.

Among all potential driving factors, SPOI was the most prominent factor that contributed to park use in both groups (q = 0.6006 in the weekday group and 0.6237 in the weekend group). Moreover, in the weekday group, the accessibility factors W-15 and D-30 had little influence (q = 0.1711 and q = 0.1933) and there was no significant difference in the other factors (q-values between 0.35 and 0.40). The park characteristics factors were slightly higher and close to 0.40. In the weekend group, the effects of the remaining significant factors were significantly different. The q-values of the three park characteristic factors (P-SI, P-TY, and P-FA) were relatively higher, while those of the four factors for accessibility and surrounding environment features were relatively close.

#### 3.2.2. The Interactive Effects of Driving Factors on Urban Park Use

This study used the interaction detector to detect 91 interaction pairs between the 13 factors in the two groups. As shown in Table 3, the synergistic effect between each pair of driving factors was manifested as bivariate enhanced or nonlinear enhanced, indicating that the interaction between the two driving factors strengthened the influence of each in-dividual factor on urban park use. Among the interactions between all factors, q(SPOI∩PSC) showed the maximum value (0.8775 in the weekday group and 0.9264 in the weekend group), indicating that the interaction between SPOI and PSC was the strongest on both weekdays and weekends. In both groups, q(P-FA∩P-TY) showed the maximum value (0.6252/0.6667) among the interactions between park characteristics factors, q(D-30∩DTC) showed the maximum value (0.7011/0.7355) among the interactions between accessibility factors, and q(SPOI∩RPD) showed the maximum value (0.7006/0.7337) among the interactions between the factors for surrounding environment features.

### 3.3. Spatial Variability in Factors Affecting Park Use via MGWR

#### 3.3.1. Model Comparison

There was heterogeneity in the geospatial relationships between the influencing factors and park use. This study constructed ordinary least squares (OLS), GWR, and MGWR models for the two groups and used the 13 independent variables from Table 1 for calibration. To test the multicollinearity of the variables used in the models, this study calculated the variance inflation factors (VIFs) for each variable using the OLS value (Table S3). The results show that the VIFs were all lower than 5, indicating no issues of multicollinearity in the models [51]. Further, Table S4 provides performance parameters for comparison with the OLS, GWR, and MGWR models. A higher adjusted R^2^ value indicates higher explanatory power and model fitness, whereas a lower Akaike information criterion value signifies model concision and a more reliable regression estimation [51]. Compared with the OLS and GWR models, the MGWR model could more accurately reflect the phenomena and was thus deemed to have the best model fit.

#### 3.3.2. Spatial Patterns of Influencing Factors

**Figure 5:**
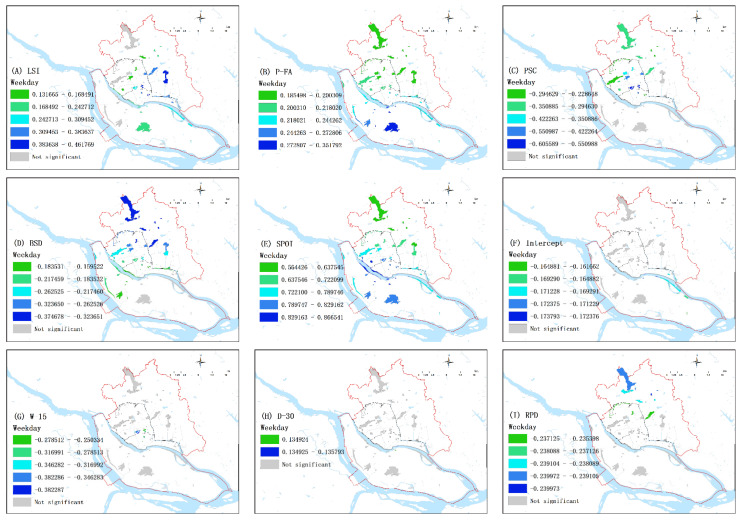

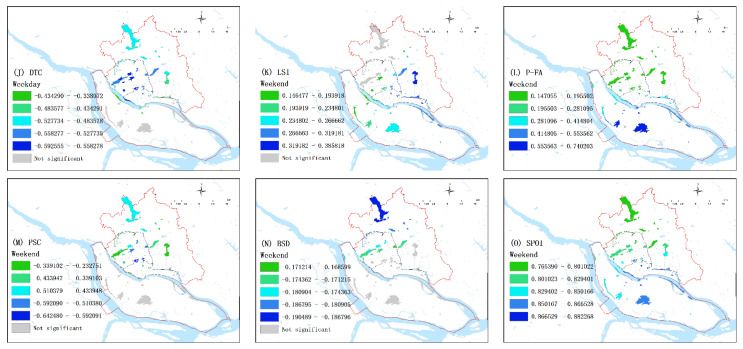
Spatial patterns of the regression coefficients of affecting factors in the weekday and weekend groups. Only significant variables (*p* < 0.01) are mapped.

This study used the MGWR model to analyze the geographic spatial relationships between the influencing factors and park use (Table 4). To reveal the spatial pattern of spatial non-stationary variables, this study only selected explanatory variables with statistical significance (*p* < 0.05) and divided each variable into five levels in terms of influencing degree using the natural breaks method. Figure 5 reveals the significant spatial differences of the influencing effects of the various explanatory variables on park use.

As shown in Table 4, SPOI had the largest coefficient mean in both groups and was globally significant and positively correlated with park use. Figure 5E,O show that the correlation coefficient increased from north to south overall, with the highest value on both sides of the Minjiang River. The south contains the new urban area, and the core office area is located along the Minjiang River, where commercial and recreational facilities are insufficient. Therefore, the increase in POIs in these areas could increase park use to the greatest extent.

P-FA was also globally and positively correlated with park use in both groups, and the correlation became stronger from north to south (Figure 5B,L). The number of parks in in the south of Cangshan District was lower than that in the north; thus, residents have limited choices. Increasing the facilities inside the parks could significantly enhance the parks’ attraction and increase the number of visitors.

The LSI, PSC, and BSD coefficients showed partial significance in the two groups (Table 4), and their spatial variation was similar. The influence of LSI increased from west to east (Figure 5A,K) because the eastern part of the city, as a newly developed area, has more parks with regular boundaries, and once these boundaries twist and turn, they can attract more visitors. PSC, based on the Dianping score, negatively affected PUD; the high value of correlation was located in the city center (Figure 5C,M) and decreased from the surrounding areas to the center. This is because Dianping often gives high scores to large landscape parks in the city center, which shows a lower dependent variable value because of the calculation rules explained in Section 3.1. This is also why P-TY is negatively correlated with park use in Table 4. BSD was also negatively correlated with park use, and its highest coefficient was located in the north of Gulou (Figure 5D,N). This is mainly because areas that contain a high density of bus stops, a large resident population, and good walking distances to parks tend to be central areas with high park density; thus, people have more options. These parks also include numerous mini-parks, which were not included in this study because they were smaller than 1600 m^2^. RPD and W-15 are also negatively correlated with park use due to the same reason of competition between large and small parks.

The intercepts (effects of location-specific factors not included in the model; 12.25%), W-15 (8.16%), D-30 (2.04%), RPD (16.33%), and DTC (74.49%) were partly significant in the weekday group (Figure 5F–J) but globally insignificant in the weekend group (Table 4). DTC was negatively associated with park use, and the effect increased closer to the city center on weekdays but not on weekends.

The P-SI, P-TY, W-SI, and WPD coefficients were globally insignificant in both groups (Table 4), which means that all parks in the study area were affected by these variables in the same ways. Meanwhile, P-SI had a 100% positive effect on weekdays but a 70.4% negative effect on weekends; this may be because, on weekends, people tend to abandon medium- and large-sized parks and drive to various scenic spots outside of the urban area for leisure. This is in part due to the convenient transportation available to urban residents. Meanwhile, small parks do not show a change. In the weekend group, W-SI was positively correlated with park use but negatively correlated with a few parks (17.35%) located along the Minjiang and Guangming Rivers, which have abundant water resources. WPD was 100% positive in the weekday group and 100% negative in the weekend group because people tend to go home rather than to the park near their workplace on weekends.

## 4. Discussion

### 4.1. The Influencing Mechanisms of Urban Park Use

Based on geographical detector analysis, this study revealed nine factors (P-SI, P-TY, P-FA, DTC, W-15, D-30, RPD, WPD, SPOI) associated with urban park use in the weekday group and ten factors (P-SI, P-TY, W-SI, P-FA, PSC, DTC, D-30, RPD, WPD, SPOI) in the weekend group. In all factors, the presence of SPOI had a dominant effect on urban park use [14,53,54], which means that promoting SPOI may increase park use most prominently. When people visit parks, they tend to also engage in other recreational activities, such as shopping, entertainment, sports, and dining. Therefore, an urban environment that provides a large number of services and facilities can attract more visitors [20]. Among the remaining significant factors, the q-values of the variables in the accessibility group were relatively lower, indicating that accessibility is not a limiting factor that affects park use in Fuzhou as it is convenient and efficient to reach Fuzhou parks. This is similar to Shenzhen, Wuhan, and some cities in Denmark [12,22,23]. However, the q-values of the variables relating to park characteristics were relatively high on weekdays and weekends, indicating that, apart from SPOI, people still paid more attention to whether a park’s type, size, facilities, services, and landscapes were attractive enough [20]. Moreover, although W-SI was not significant on weekdays, which contradicts some studies [16,17,55,56], this was due to the abundant rivers and lakes in Fuzhou’s urban parks, and no obvious variations existed among the parks. Thus, this study is unable to assert whether water is an important feature of a park.

Further investigation showed that the interaction effect between each pair of drivers manifested as bivariate enhancement or nonlinear enhancement. From another perspective, the interaction of the two driving factors enhanced the influence of each factor on urban park use. Therefore, urban planners and park managers should improve urban parks from multiple dimensions to enhance their utilization efficiency.

This study also used MGWR to detect the spatial variation of regression coefficients in the influence of various variables on park use at different scales and found that different locations in the study area had different responses to some influencing factors, which suggests that planners should consider different locations of the city for specific research. In the north of the study area is the old town, which is rich in services and facilities; thus, the importance of SPOI and P-FA was diminished. In the south, the opposite was true. In the middle of the study area, the strongest effect of DTC on PUD was found (weekday group), indicating that the closer to the city center the park is, the greater the demand for park use. This is possibly due to the faster, busier pace of urban life that leads people to seek solace and relaxation by visiting parks. However, the DTC is insignificant in the south, which is far from the city center. This suggests that more consideration should be given to adding more parks in the areas closer to the city center. If there are no large areas available, small areas, such as pocket parks, can be added [57]. Although LSI was not significant in the geographical detector, it had a high influence coefficient in the east, indicating that a rich boundary design in these areas would help increase park use. Moreover, BSD, RPD, and W-15 were negatively correlated with park use [23], contrary to some previous studies [14,15,17]. As explained above, this was due to the competition among multiple parks in areas with perfect infrastructure; however, the specific quantitative relationship should be explored in future studies. Simultaneously, this result also indicates that, since the launch of the “Greening Fuzhou” project in 2016, the supply of Fuzhou parks is becoming more adequate overall, though whether the distribution of parks in each part is equal or fair is unknown. In the future, we can consider measuring the supply and demand of parks from the perspective of their actual use to study the fairness and justice of urban green space environment distribution.

Finally, by comparing the results of the pairwise model series for weekdays and weekends, this study found that the types and mechanisms of factors affecting park use were the same in the two groups. However, some small differences could indicate some noticeable effects [58]. For example, in all three regression models in Section 3.3, the adjusted R^2^ values for the weekend group were lower than those for the weekday group, suggesting that weekend park visits were affected by more complex factors that were not measured by the available independent variables. The q-values of SPOI, D-30, PSC, W-SI, and P-SI in the weekend group were higher than those in the weekday group, while those of P-FA, DTC, W-15, RPD, and WPD in the weekday group were higher than those in the weekend group, indicating that factors reflecting subjective preference had a strong predictive effect on weekend visitors. However, factors reflecting convenience had a strong predictive effect on weekday visitors.

### 4.2. Methodological and Theoretical Contributions

The previous studies on urban park use have adopted traditional survey methods that are not only time- and energy-consuming, but also make it difficult to measure urban park use per hour at the city level. Some studies have used traditional geospatial data, such as social media platforms and mobile phone signaling, but have been unable to accurately measure the use of urban parks due to poor resolution or data scales [59]. Accordingly, this study used RTUD data to provide a more convenient, feasible, and reproducible innovative method for data collection and urban park use measurement at the city level. Regarding the discussion on the influence mechanism of parks, contrary to previous studies, which were inaccurate due to their neglect of the geospatial effect, this study comprehensively used geographic detectors and MGWR, proposed an impact research model based on a geospatial method, quantified the individual and interactive effects of various influencing factors on urban park use, and confirmed the spatial heterogeneity of influencing factors. Thus, thus study enriches and deepens theoretical research in this field. The method proposed herein can be used as a model to evaluate urban park use and can also be applied to other similar large-scale social studies. The confirmation of the spatial heterogeneity of influencing factors inspires us to develop further understanding of the use of urban parks from diverse perspectives in our theoretical research.

### 4.3. Implications for Urban Park Planning and Management in Similar Cities

First, better land use policies can be developed by considering the external factors that affect urban park use when selecting sites for park development [60,61]. As SPOI dominates both weekend and weekday park use, it is important to locate new urban parks close to recreational business districts [14], especially in areas where additional urban parks are needed.

Second, the interaction between various influencing factors can promote park use from multiple dimensions. According to the results, this study suggests building an urban park complex, realizing the complementarity of commercial and service facilities inside and outside the parks and improving the service levels in and around the parks. Moreover, P-TY and P-FA should be kept in sync, for example, by including a corresponding community communication square and morning exercise equipment in community parks to maximize the benefits of the park characteristic factors group. Similarly, the 30 min car accessibility time should be optimized according to location, and the supply of service facilities related to residents’ daily lives should be improved around the parks in residential areas with larger populations [14].

Third, in view of the spatial heterogeneity of the influencing factors of park use, this study suggests that different measures should be taken for urban park construction in different locations. For example, in places with low SPOI density, public service facilities (restaurants, public toilets, coffee bars, galleries, sports venues, etc.) can be added in the parks to improve the construction of urban commercial facilities [13]. Further, areas strongly affected by DTC should be delimited, and the supply of green park space should be increased as much as possible in these places to meet residents’ needs. In new urban areas with dull park boundaries, such boundaries can be optimized by setting squares, adopting iconic landscape sketches, and the use of hierarchical plant landscaping to increase contact areas with the city [23] and attract more park visitors. Moreover, for the weekday group, increasing park area can positively correlate with park use; however, as this can be difficult, this study suggests paying attention to and repairing the city’s old parks and those in disrepair [14]. For the weekend group, P-SI was mostly negatively correlated with park use, which indicates that, when considering the actual recreation services of urban parks, a bigger park area is not necessarily better [13]. Small but excellent parks could be built in appropriate locations to provide abundant recreation services.

Finally, when planning and constructing parks, there is a need to consider both the convenient use of parks on weekdays and visitors’ subjective preference for leisure use on weekends. Mini-parks with squares and rest facilities can be established in places with dense populations and convenient transportation. Simultaneously, we can make full use of the park landscapes, create a variety of large parks with distinctive characteristics and comprehensive services, and provide more opportunities for people to choose parks according to their preferences on weekends.

### 4.4. Limitations

First, this exploratory study used RTUD data to evaluate urban park use from a geospatial perspective. However, to date, RTUD data are only applicable to China; thus, the methodology proposed herein is only applicable to urban parks in China. Similar data will undoubtedly be developed in other countries in the future. Second, RTUD data could only provide the total number of users in each area unit, while individual sociodemographic attributes that affect park use, such as user age, gender, and income status, were not available. Data on use characteristics, such as the frequency and duration of individuals’ park use, were also unavailable. Meanwhile, socioeconomic factors such as per capita GDP may also have a strong explanatory power in the model. However, as these data can currently only be obtained at the district level in China, they were outside this study’s scope. These data limitations restrict the reach of our study. Third, the RTUD data used herein were collected during one month in the fall season and do not reflect park use during other seasons. Finally, although RTUD data are the most accurate population data with a resolution of 25 m, this study was unable to include a large number of mini-parks smaller than 1600 m^2^, and the complex competitive relationship between mini-parks and large parks could be why some of this study’s results were contrary to previous studies.

As such, further research can utilize this study’s methods and results to compare the use of Fuzhou parks in different seasons. Further, the combination of drone photography [62] or Wi-Fi probe identification [63] and questionnaires [24] could be used to obtain data related to sociodemographic attribute variables to better understand people’s real behavior in parks and improve the influencing factors of park use. Meanwhile, if per capita GDP data to enhance the accuracy of this study’s findings still cannot be obtained, alternative data could be considered. Finally, future research could develop a set of methods to determine the number of users of mini-parks that are less than 1600 m^2^ and quantify the competitive relationship between large and small parks, thus gaining a deeper understanding of urban park use.

## 5. Conclusions

Urban parks provide places for city residents to engage in sports and leisure activities, resulting in substantial health and well-being benefits. However, the extent to which these benefits are realized highly depends on a thorough understanding of how much urban parks are used. Based on the geospatial perspective, this study used RTUD data to calculate urban park use and introduced the comprehensive use of geographic detectors and MGWR to explore the determinants of relevant factors on urban park use, their interaction effects, and the influencing degree of spatial changes.

The results show that the PUD of small parks in the city center was high, so attention should be paid to the role of small parks in the urban green space system. The geographical detectors’ results indicate that most of the variables related to park characteristics, accessibility, and surrounding environment features were significantly correlated with urban park use, while SPOI had the greatest impact on urban park use, which indicates that increasing commercial and recreational facilities and service levels around parks could be the best way to attract more visitors. The interaction effect of two drivers was higher than that of a single driver, and the interaction effect between SPOI and PSC had the greatest impact on urban park use. The MGWR results show that the spatial changes of many influencing factors, such as SPOI, P-FA, LSI, and DTC, were globally or locally significant, indicating that different locations in the study area were affected by different factors to different degrees. This suggests the need to adopt zoning when constructing parks at the city level to coordinate the overall development and utilization of parks in the main urban area. Finally, people chose to visit parks based on convenience on weekdays and according to their subjective preference on weekends, which suggests the need to build both convenient small parks and characteristic large parks.

The results can explain residents’ urban park use in Fuzhou City, while confirmation of the spatial heterogeneity of the influencing factors enriches current theoretical research on park use. The models and methods used in this study can be further verified in similar cities to seek the best way to optimize park construction and enhance parks’ benefits. Further research should include mini-parks that cannot be measured by current RTUD data and should obtain data related to sociodemographic and socioeconomic attribute variables and park use data in different seasons to deepen research into the mechanisms affecting urban park use.

## Figures and Tables

**Table 1:** Variables: description and data sources.

Variable Type	Index		Description	Unit	Data Source
Dependent variable	Park user density (PUD)		Average active Tencent users per hour per unit area during peak visits on weekdays and weekends	Population/ha·h	Collected from RUTD
Independent variable	Park characteristics	Park size (P-SI)	Park area	m^2^	Extracted from AMAP
		Park type (P-TY)	Comprehensive parks, special parks, community parks, mini-parks	-	Comprehensive parks (=7), special parks (=5), community parks (=3), mini-parks (=1)
		Landscape shape index (LSI)	The landscape shape index	-	$\mathrm{LSI}=2\frac{\sqrt{\pi \times Si}}{Ci}$
		Water size (W-SI)	Water area	m^2^	Extracted from AMAP
		Park facilities (P-FA)	The density of the number of park facilities, such as playgrounds, themed plazas, lounge corridors, restaurants, shops, toilets, and parking lots	n/ha	Field research
		Park service capability (PSC)	The overall park rating from the perspectives of scenery, hygiene, safety, and other services	Grade	The average scores from comments on Dianping were calculated (www.dianping.com, accessed on 7 December 2021)
	Accessibility	Distance to the city center (DTC)	The distance from the park to the city center	m	The network distance from the park centroid to Fuzhou Wuyi Square was calculated in ArcGIS 10.6
		Bus station density (BSD)	The density of the number of bus stations within buffer areas of each park	n/ha	Calculated in ArcGIS 10.6, buffer analysis was conducted based on data from the Fuzhou public transportation website (http://fuzhou.gongjiao.com/, accessed on 12 November 2021)
		Walking in an isochronous circle (15 min) (W-15)	Area accessibility from walking for 15 min in non-peak hours on weekdays from the park	m^2^	Used the AMAP real-time path planning tool to obtain a grid file describing the time distance to the park in ArcGIS 10.6
		Driving in an isochronous circle (30 min) (D-30)	Area accessibility from driving for 30 min in non-peak hours on weekdays from the park	m^2^	Used the AMAP real-time path planning tool to obtain a grid file describing the time distance to the park in ArcGIS 10.6
	Surrounding environment features	Residential population density (RPD)	The density of the residential population within the buffer areas of each park	Population/ha	Calculated in ArcGIS 10.6, buffer analysis was conducted based on mobile phone signaling data
		Working population density (WPD)	The density of the working population within the buffer areas of each park	Population/ha	Calculated in ArcGIS 10.6, buffer analysis was conducted based on mobile phone signaling data
		Park-surrounding facilities and services (SPOI)	The density of the number of SPOI within the buffer areas of each park	n/ha	Calculated in ArcGIS 10.6, buffer analysis was conducted based on data from AMAP Map in 2021

**Table 2:** Results from factor detector analysis between urban park use and the affecting factors (A) Weekday; (B) Weekend.

(A) Weekday													
Factor	P-SI	P-TY	LSI	W-SI	P-FA	PSC	DTC	BSD	W-15	D-30	RPD	WPD	SPOI
q-statistic	0.3813	0.3928	0.0537	0.1177	0.3993	0.1240	0.3984	0.1347	0.1711	0.1933	0.3506	0.3619	0.6006
*p*-value	0	0	0.61	0.09	0	0.15	0	0.09	0.01	0.02	0	0	0
**(B) Weekend**													
**Factor**	**P-SI**	**P-TY**	**LSI**	**W-SI**	**P-FA**	**PSC**	**DTC**	**BSD**	**W-15**	**D-30**	**RPD**	**WPD**	**SPOI**
q-statistic	0.4500	0.4048	0.0482	0.1396	0.3655	0.1666	0.3131	0.1405	0.1266	0.2475	0.3191	0.2346	0.6237
*p*-value	0	0	0.66	0.03	0	0.05	0	0.07	0.09	0	0	0	0

Note: P-SI: park size; P-TY: park type; LSI: landscape shape index; W-SI: water size; P-FA: park facilities; PSC: park service capability; DTC: distance to the city center; BSD: bus station density; W-15: 15 min of walking; D-30: 30 min of driving; RPD: residential population density; WPD: working population density; SPOI: park-surrounding facilities and services.

**Table 3:** Interaction detector analysis of driving factors on urban park use (A) Weekday; (B) Weekend.

(A) Weekday													
	P-SI	P-TY	LSI	W-SI	P-FA	PSC	DTC	BSD	W-15	D-30	RPD	WPD	SPOI
P-SI	0.3813												
P-TY	0.5029 (EB)	0.3928											
LSI	0.4299 (EB)	0.4660 (EN)	0.0537										
W-SI	0.4003 (EB)	0.4373 (EB)	0.2000 (EN)	0.1177									
P-FA	0.6126 (EB)	0.6252 (EB)	0.5830 (EN)	0.4807 (EB)	0.3993								
PSC	0.5469 (EN)	0.5831 (EN)	0.3293 (EN)	0.1992 (EB)	0.5710 (EN)	0.1240							
DTC	0.6668 (EB)	0.6863 (EB)	0.4879 (EN)	0.5143 (EB)	0.6474 (EB)	0.7198 (EN)	0.3984						
BSD	0.5232 (EB)	0.5785 (EN)	0.3725 (EN)	0.2998 (EN)	0.6617 (EN)	0.5675 (EN)	0.6109 (EN)	0.1347					
W-15	0.6291 (EN)	0.7041 (EN)	0.4241 (EN)	0.3103 (EN)	0.6816 (EN)	0.4635 (EN)	0.6357 (EN)	0.5330 (EN)	0.1711				
D-30	0.6120 (EN)	0.6328 (EN)	0.4105 (EN)	0.3380 (EN)	0.5577 (EB)	0.4452 (EN)	0.7011 (EN)	0.5354 (EN)	0.6188 (EN)	0.1933			
RPD	0.7217 (EB)	0.6477 (EB)	0.5035 (EN)	0.4752 (EB)	0.7150 (EB)	0.7177 (EN)	0.6075 (EB)	0.6360 (EN)	0.6509 (EN)	0.5808 (EN)	0.3506		
WPD	0.7438 (EB)	0.7239 (EB)	0.5550 (EN)	0.4466 (EB)	0.7120 (EB)	0.6401 (EN)	0.5402 (EB)	0.6833 (EN)	0.5948 (EN)	0.6145 (EN)	0.5819 (EB)	0.3619	
SPOI	0.8355 (EB)	0.8455 (EB)	0.7965 (EN)	0.7143 (EB)	0.7885 (EB)	0.8775 (EN)	0.7031 (EB)	0.7551 (EN)	0.7118 (EB)	0.7481 (EB)	0.7006 (EB)	0.6978 (EB)	0.6006
**(B) Weekend**													
	**P-SI**	**P-TY**	**LSI**	**W-SI**	**P-FA**	**PSC**	**DTC**	**BSD**	**W-15**	**D-30**	**RPD**	**WPD**	**SPOI**
P-SI	0.4500												
P-TY	0.5584 (EB)	0.4048											
LSI	0.4928 (EB)	0.4904 (EN)	0.0482										
W-SI	0.4746 (EB)	0.4534 (EB)	0.2106 (EN)	0.1396									
P-FA	0.6163 (EB)	0.6667 (EB)	0.5739 (EN)	0.4594 (EB)	0.3655								
PSC	0.6077 (EB)	0.6510 (EN)	0.3376 (EN)	0.2345 (EB)	0.5385 (EB)	0.1666							
DTC	0.6211 (EB)	0.6019 (EB)	0.3935 (EN)	0.4376 (EB)	0.6075 (EB)	0.7031 (EN)	0.3131						
BSD	0.5710 (EB)	0.5941 (EN)	0.3581 (EN)	0.3026 (EN)	0.6742 (EN)	0.4866 (EN)	0.5373 (EN)	0.1405					
W-15	0.5884 (EB)	0.6601 (EN)	0.3653 (EN)	0.2724 (EB)	0.6546 (EN)	0.4662 (EN)	0.5677 (EN)	0.4426 (EN)	0.1266				
D-30	0.7872 (EN)	0.7724 (EN)	0.4477 (EN)	0.4397 (EN)	0.5805 (EB)	0.5148 (EN)	0.7355 (EN)	0.5346 (EN)	0.6249 (EN)	0.2475			
RPD	0.7472 (EB)	0.6118 (EB)	0.4926 (EN)	0.4521 (EB)	0.6516 (EB)	0.7518 (EN)	0.5481 (EB)	0.5802 (EN)	0.6584 (EN)	0.6304 (EN)	0.3191		
WPD	0.6854 (EB)	0.6370 (EB)	0.4091 (EN)	0.3523 (EB)	0.6199 (EN)	0.5856 (EN)	0.4156 (EB)	0.6054 (EN)	0.4960 (EN)	0.5998 (EN)	0.5372 (EB)	0.2346	
SPOI	0.8752 (EB)	0.8509 (EB)	0.8206 (EN)	0.7185 (EB)	0.8165 (EB)	0.9264 (EN)	0.7736 (EB)	0.7703 (EB)	0.7428 (EB)	0.7611 (EB)	0.7337 (EB)	0.7134 (EB)	0.6237

Note: P-SI: park size; P-TY: park type; LSI: landscape shape index; W-SI: water size; P-FA: park facilities; PSC: park service capability; DTC: distance to the city center; BSD: bus station density; W-15: 15 min of walking; D-30: 30 min of driving; RPD: residential population density; WPD: working population density; SPOI: park-surrounding facilities and services. EN represents the nonlinear enhancement of two factors, and EB represents the binary enhancement of two factors (see Table S2).

**Table 4:** Regression results of MGWR model.

Variable	Weekday				Weekend			
	Coefficient	*t*-Test			Coefficient	*t*-Test		
	Mean	*p* < 0.05 (%)	Positive Value (%)	Negative Value (%)	Mean	*p* < 0.05(%)	Positive Value (%)	Negative Value (%)
Intercept	−0.145	12.25	0	100	0.013	0	82.65	17.35
P-SI	0.035	0	100	0	−0.005	0	29.60	70.40
P-TY	−0.120	0	0	100	−0.052	0	0	100
LSI	0.240	68.37	100	0	0.234	78.57	100	0
W-SI	0.015	0	100	0	0.016	0	82.65	17.35
P-FA	0.223	100	100	0	0.312	100	100	0
PSC	−0.230	46.94	10.2	89.80	−0.316	54.08	0	100
DTC	−0.414	74.49	2.04	97.96	−0.035	0	12.24	87.76
BSD	−0.225	83.67	0	100	−0.154	36.73	0	100
W-15	−0.119	8.16	6.12	93.88	−0.097	0	0	100
D-30	0.115	2.04	100	0	0.094	0	100	0
RPD	−0.223	16.33	0	100	−0.044	0	0	100
WPD	0.082	0	100	0	−0.039	0	0	100
SPOI	0.761	100	100	0	0.838	100	100	0

Note: P-SI: park size; P-TY: park type; LSI: landscape shape index; W-SI: water size; P-FA: park facilities; PSC: park service capability; DTC: distance to the city center; BSD: bus station density; W-15: 15 min of walking; D-30: 30 min of driving; RPD: residential population density; WPD: working population density; SPOI: park-surrounding facilities and services.

## Data Availability

Data will be made available on request.

## Supplementary Materials

The following supporting information can be downloaded at: https://www.mdpi.com/article/10.3390/ijerph20054237/s1, Figure S1: Hourly RTUD data on three consecutive Wednesdays of parks in the study area; Figure S2: Hourly RTUD data on three consecutive Saturdays of parks in the study area; Figure S3: Interaction detection. Note: q(X) and q(X1) are calculated respectively. Overlay the two layers X1 and X2 to get the new layer X1nX2, calculate q(X1∩Y2). Judge the interaction type of the two factors according to Table S2; Table S1: Results of repeatable multivariate analysis of variance; Table S2: The interactive types of two factors and the interactive relationship; Table S3: The variance inflation factors (VIFs) for each variable by OLS; Table S4: Model fit metrics for ordinary least squares (OLS) regression, geographically weighted regression (GWR), and multiscale geographically weighted regression (MGWR).

## Abbreviations

RTUD, real-time Tencent user density; SPOI, park-surrounding facilities and services; P-SI, park size; P-TY, park type; PSC, park service capacity; P-FA, park facilities; PUD, park use density; LSI, landscape shape index; DTC, distance to the city center; W-SI, water size; BSD, bus station density; RPD, resident population density; WPD, working population density; POI, point of interest; W-15, walking for 15 min in an isochronous circle; D-30, driving for 30 min in an isochronous circle.
