# Supplementary material for: Exploring Factors Affecting Urban Park Use from a Geospatial Perspective: A Big Data Study in Fuzhou, China
Source: Int J Environ Res Public Health. 2023 Feb 27;20(5):4237. doi: 10.3390/ijerph20054237 (PMC10002407; doi:10.3390/ijerph20054237)
Supplement: Supplementary file 1 [file ijerph-20-04237-s001.zip › ijerph-2086507-supplementary/Supplementary_material__tables_.pdf]

Exploring factors affecting urban park use from a geospatial perspective: A big data study in Fuzhou, China

Supplementary Tables

Table S1 Results of repeatable multivariate analysis of variance.

differences between the source	SS	df	MS	F	P-value	F crit
between parks	353197.5	1	353197.5	1804.469	0	3.86529
between weekdays and weekends	1418774	97	14626.54	74.72628	0	1.287376
interaction	370361.8	97	3818.163	19.50681	0	1.287376
interior	76728.06	392	195.7348			
total	2219061	587				

Table S2 The interactive types of two factors and the interactive relationship.

Description	lnteraction
$q(x_1 \cap x_2) < Min(q(x_1), q(x_2))$	Weakened, nonlinear
$Min(q(x_1), q(x_2)) < q(x_1 \cap x_2) < Max(q(x_1), q(x_2))$	Weakened, unique
$q(x_1 \cap x_2) > Max(q(x_1), q(x_2))$	Enhanced, bilinear
$q(x_1 \cap x_2) = q(x_1) + q(x_2)$	Independent
$q(x_1 \cap x_2) > q(x_1) + q(x_2)$	Enhanced, nonlinear

Table S3 The variance inflation factors (VIFs) for each variable by OLS.

(A)weekday

Variable	P-si	P-ty	Lsi	W-si	P-fa	Psc	Dtc	Bsd	W-15	D-30	Rpd	Wpd	Spoi
VIF	1.667	1.880	1.208	1.583	1.227	1.649	2.583	1.734	2.070	1.337	3.466	3.101	4.517

(B)weekend

Variable	P-si	P-ty	Lsi	W-si	P-fa	Psc	Dtc	Bsd	W-15	D-30	Rpd	Wpd	Spoi
VIF	1.667	1.880	1.208	1.583	1.227	1.649	2.583	1.734	2.070	1.337	3.466	3.101	4.517

Table S4 Model fit metrics for ordinary least squares (OLS) regression, geographicallyweighted regression (GWR), and multiscale geographically weighted regression(MGWR).

park use	Fit metrics	Model		
		OLS	GWR	MGWR
weekday	R2(adjust)	0.640755	0.618797	0.664
	AICc	820.287	829.944	206.367
weekend	R2(adjust)	0.603265	0.577156	0.65
	AICc	843.8697	854.3444	210.292
