# Supplementary material for: Exploring Factors Affecting Urban Park Use from a Geospatial Perspective: A Big Data Study in Fuzhou, China
Source: Int J Environ Res Public Health. 2023 Feb 27;20(5):4237. doi: 10.3390/ijerph20054237 (PMC10002407; doi:10.3390/ijerph20054237)
Supplement: Supplementary file 1 [file ijerph-20-04237-s001.zip › ijerph-2086507-supplementary/Supplementary_material_figures _.pdf]

Exploring factors affecting urban park use from a geospatial perspective: A big data study in Fuzhou, China

Supplementary Figures

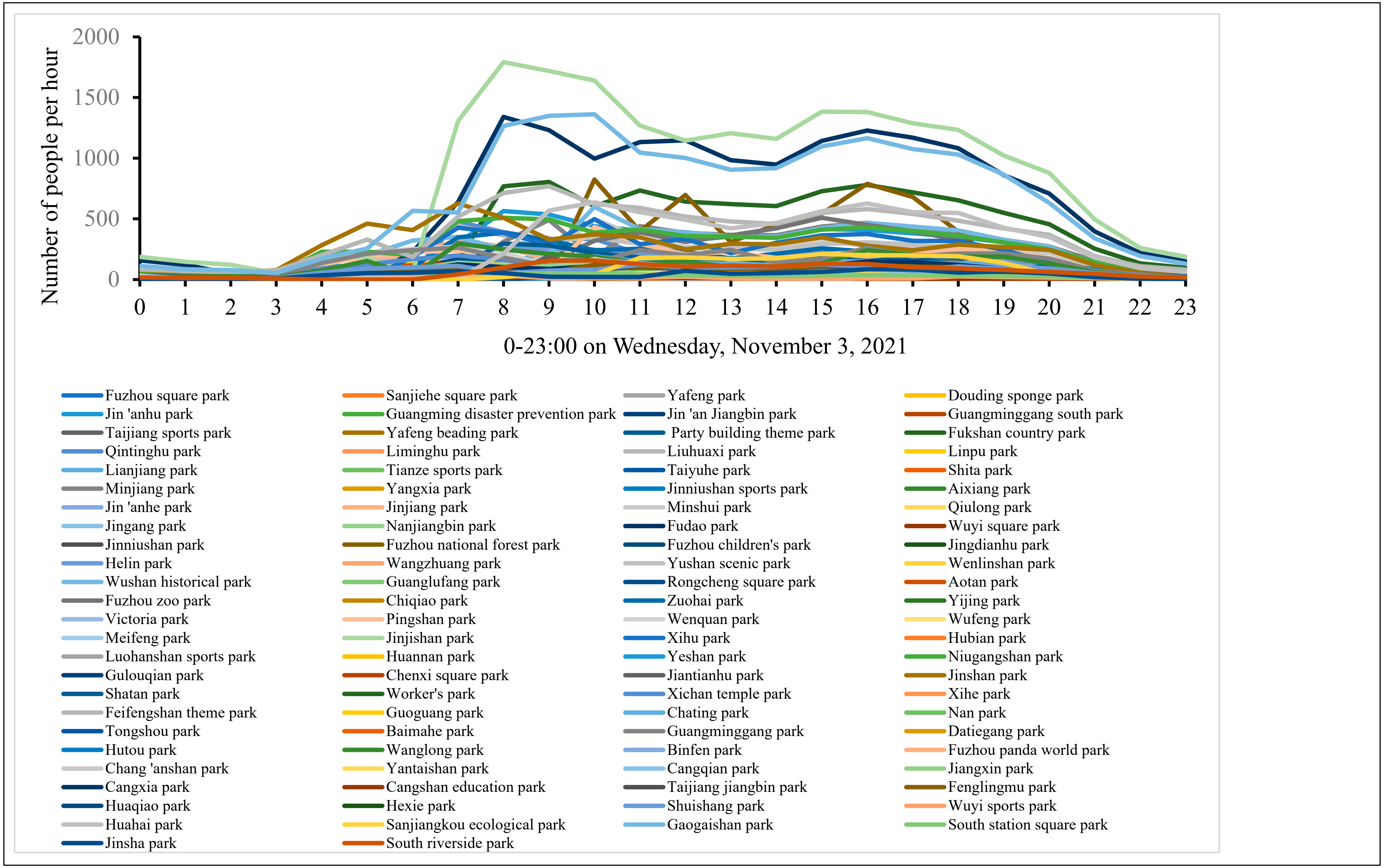

Number of people per hour
2000
1500
1000
500
0
0 1 2 3 4 5 6 7 8 9 10 11 12 13 14 15 16 17 18 19 20 21 22 23
0-23:00 on Wednesday, November 3, 2021
Fuzhou square park
Sanjiehe square park
Yafeng park
Douding sponge park
Jin 'anhu park
Guangming disaster prevention park
Jin 'an Jiangbin park
Guangminggang south park
Taijiang sports park
Yafeng beading park
Party building theme park
Fukshan country park
Qintinghu park
Liminghu park
Liuhuaxi park
Linpu park
Lianjiang park
Tianze sports park
Taiyuhe park
Shita park
Minjiang park
Yangxia park
Jinniushan sports park
Aixiang park
Jin 'anhe park
Jinjiang park
Minshui park
Qiulong park
Jingang park
Nanjiangbin park
Fudao park
Wuyi square park
Jinniushan park
Fuzhou national forest park
Fuzhou children's park
Jingdianhu park
Helin park
Wangzhuang park
Yushan scenic park
Wenlinshan park
Wushan historical park
Guanglufang park
Rongcheng square park
Aotan park
Fuzhou zoo park
Chiqiao park
Zuohai park
Yijing park
Victoria park
Pingshan park
Wenquan park
Wufeng park
Meifeng park
Jinjishan park
Xihu park
Hubian park
Luohanshan sports park
Huannan park
Yeshan park
Niugangshan park
Gulouqian park
Chenxi square park
Jiantianhu park
Jinshan park
Shatan park
Worker's park
Xichan temple park
Xihe park
Feifengshan theme park
Guoguang park
Chating park
Nan park
Tongshou park
Baimahe park
Guangminggang park
Datiegang park
Hutou park
Wanglong park
Binfen park
Fuzhou panda world park
Chang 'anshan park
Yantaishan park
Cangqian park
Jiangxin park
Cangxia park
Cangshan education park
Taijiang jiangbin park
Fenglingmu park
Huaqiao park
Hexie park
Shuishang park
Wuyi sports park
Huahai park
Sanjiangkou ecological park
Gaogaishan park
South station square park
Jinsha park
South riverside park

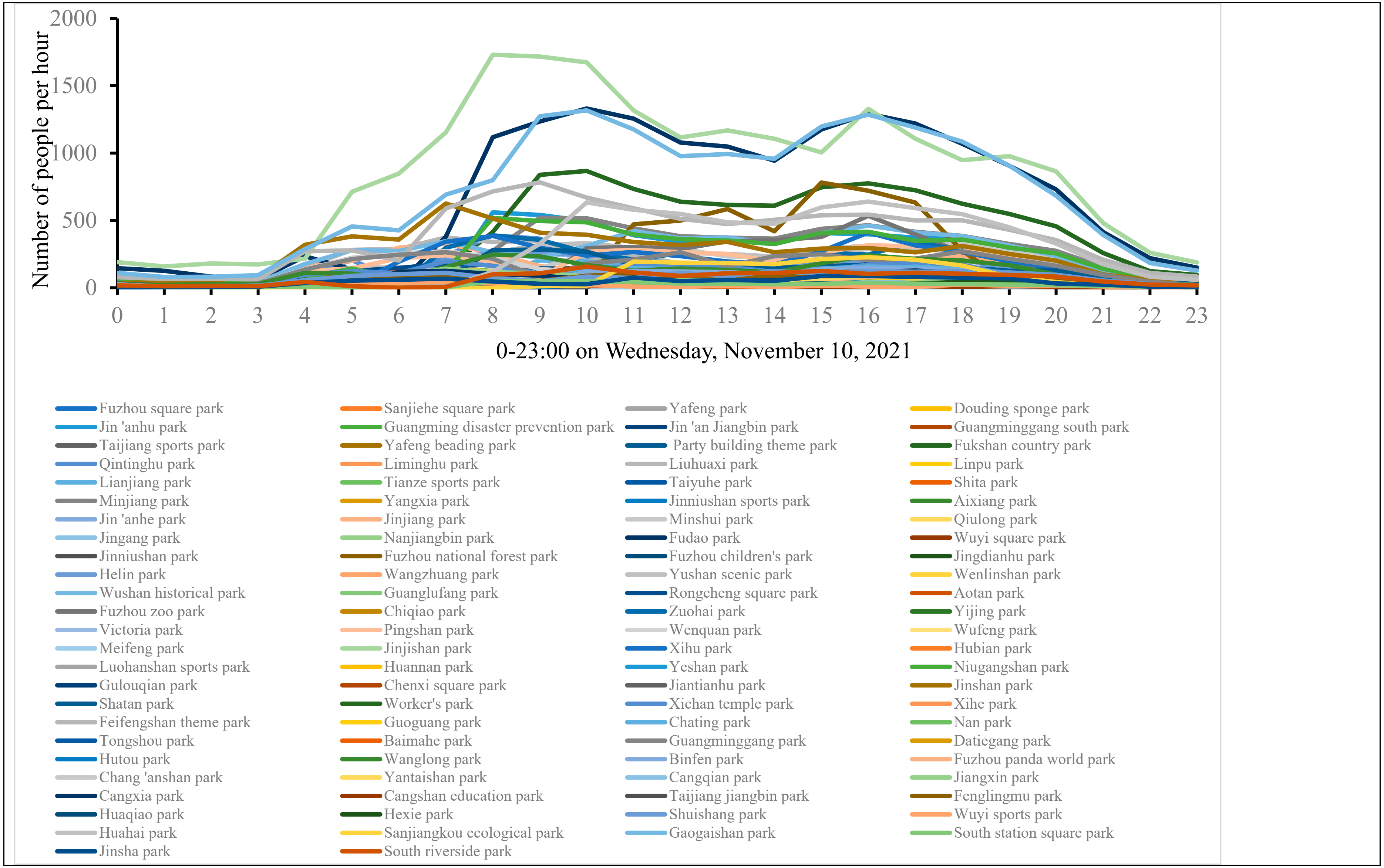
Number of people per hour
2000
1500
1000
500
0
0 1 2 3 4 5 6 7 8 9 10 11 12 13 14 15 16 17 18 19 20 21 22 23
0-23:00 on Wednesday, November 10, 2021
Fuzhou square park
Jin 'anhu park
Taijiang sports park
Qintinghu park
Lianjiang park
Minjiang park
Jin 'anhe park
Jingang park
Jinniushan park
Helin park
Wushan historical park
Fuzhou zoo park
Victoria park
Meifeng park
Luohanshan sports park
Gulouqian park
Shatan park
Feifengshan theme park
Tongshou park
Hutou park
Chang 'anshan park
Cangxia park
Huaqiao park
Huahai park
Jinsha park
Sanjiehe square park
Guangming disaster prevention park
Yafeng beading park
Liminghu park
Tianze sports park
Yangxia park
Jinjiang park
Nanjiangbin park
Fuzhou national forest park
Wangzhuang park
Guanglufang park
Chiqiao park
Pingshan park
Jinjishan park
Huannan park
Chenxi square park
Worker's park
Guoguang park
Baimahe park
Wanglong park
Yantaishan park
Cangshan education park
Hexie park
Sanjiangkou ecological park
South riverside park
Yafeng park
Jin 'an Jiangbin park
Party building theme park
Liuhuaxi park
Taiyuhe park
Jinniushan sports park
Minshui park
Fudao park
Fuzhou children's park
Yushan scenic park
Rongcheng square park
Zuohai park
Wenquan park
Xihu park
Yeshan park
Jiantianhu park
Xichan temple park
Chating park
Guangminggang park
Binfen park
Cangqian park
Taijiang jiangbin park
Shuishang park
Gaogaishan park
Douding sponge park
Guangminggang south park
Fukshan country park
Linpu park
Shita park
Aixiang park
Qiulong park
Wuyi square park
Jingdianhu park
Wenlinshan park
Aotan park
Yijing park
Wufeng park
Hubian park
Niugangshan park
Jinshan park
Xihe park
Nan park
Datiegang park
Fuzhou panda world park
Jiangxin park
Fenglingmu park
Wuyi sports park
South station square park

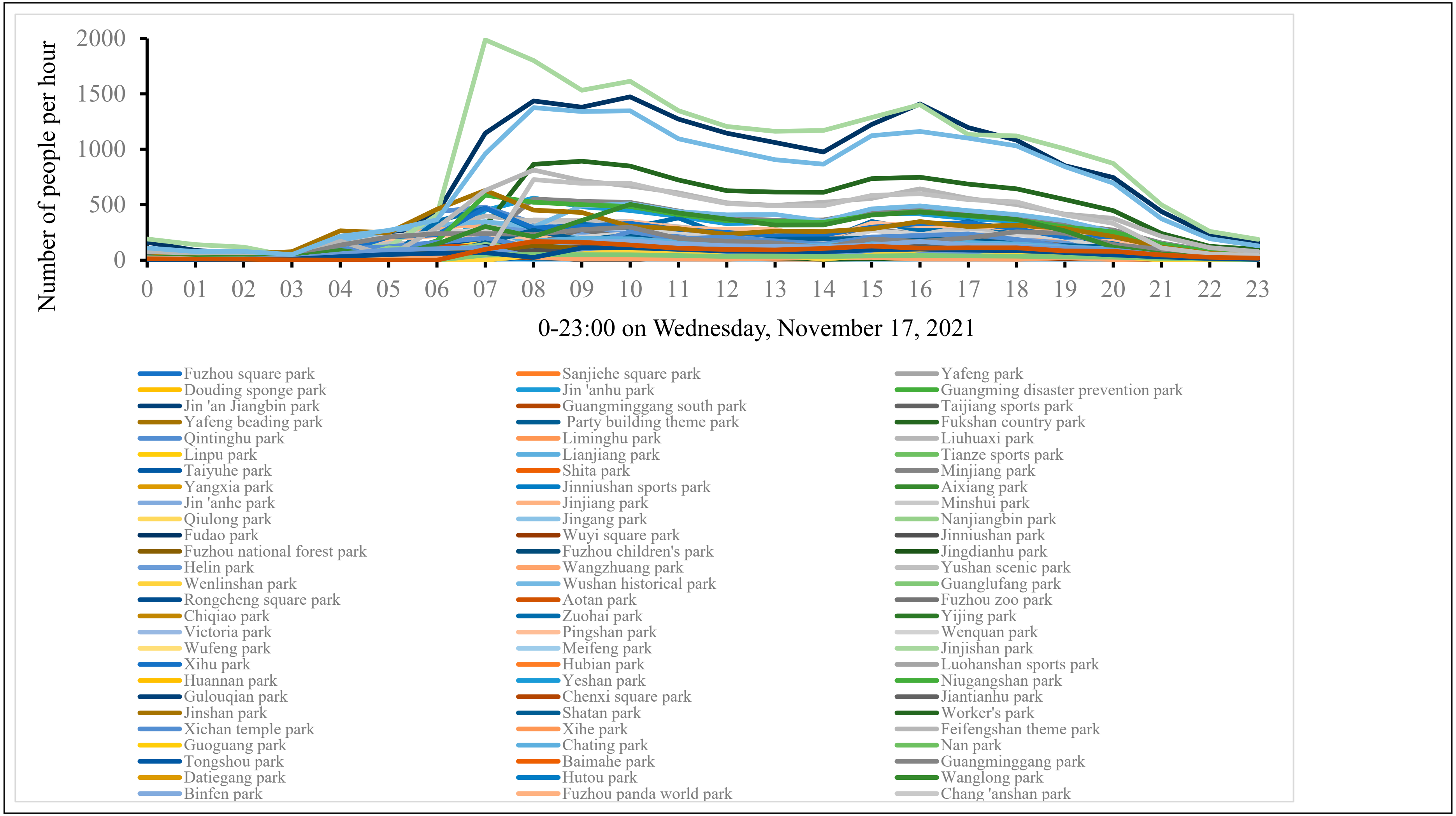

Figure S1 Hourly RTUD data on three consecutive Wednesdays of parks in the study area

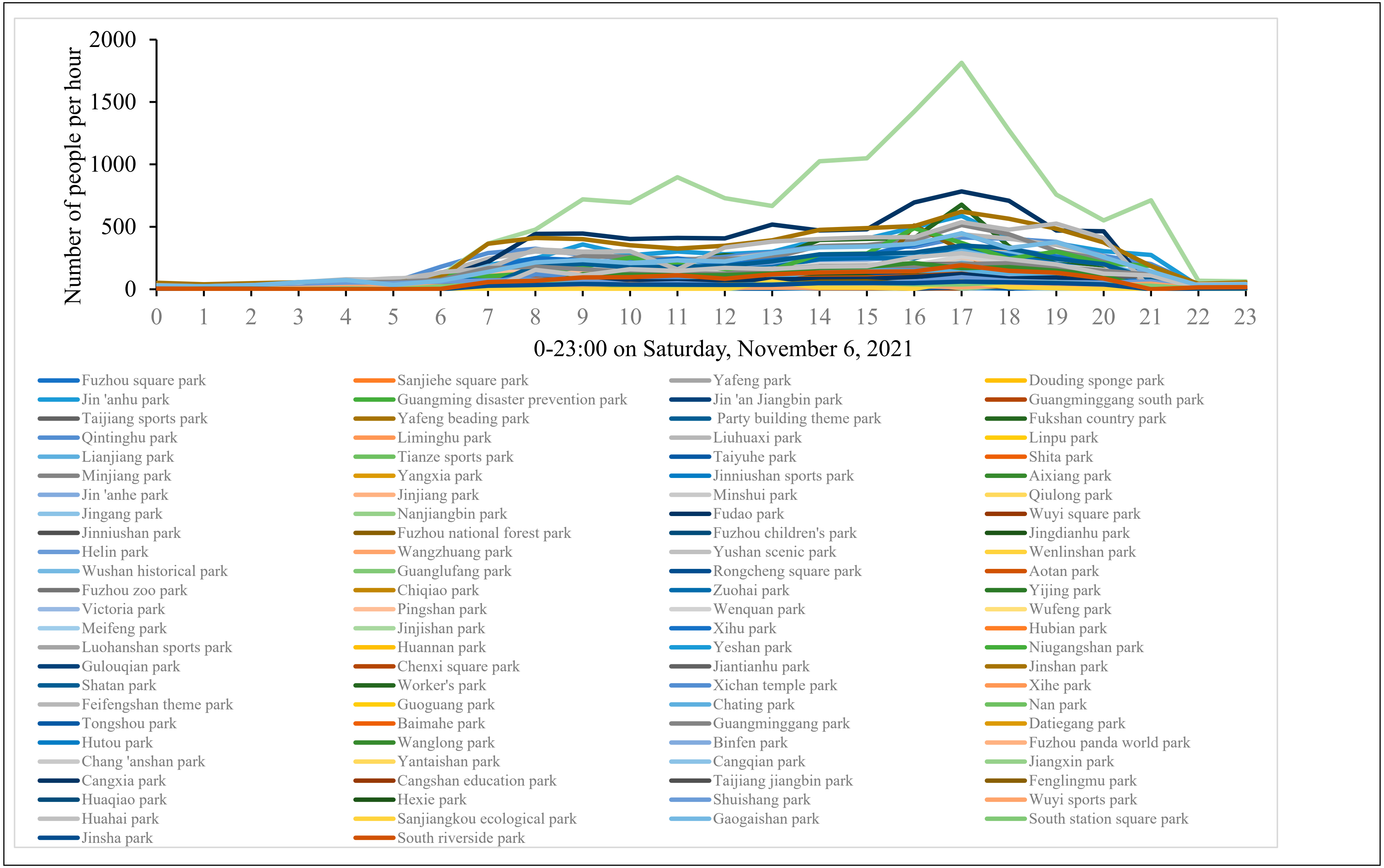
Number of people per hour
2000
1500
1000
500
0
0 1 2 3 4 5 6 7 8 9 10 11 12 13 14 15 16 17 18 19 20 21 22 23
0-23:00 on Saturday, November 6, 2021
Fuzhou square park
Jin 'anhu park
Taijiang sports park
Qintinghu park
Lianjiang park
Minjiang park
Jin 'anhe park
Jingang park
Jinniushan park
Helin park
Wushan historical park
Fuzhou zoo park
Victoria park
Meifeng park
Luohanshan sports park
Gulouqian park
Shatan park
Feifengshan theme park
Tongshou park
Hutou park
Chang 'anshan park
Cangxia park
Huaqiao park
Huahai park
Jinsha park
Sanjiehe square park
Guangming disaster prevention park
Yafeng beading park
Liminghu park
Tianze sports park
Yangxia park
Jinjiang park
Nanjiangbin park
Fuzhou national forest park
Wangzhuang park
Guanglufang park
Chiqiao park
Pingshan park
Jinjishan park
Huannan park
Chenxi square park
Worker's park
Guoguang park
Baimahe park
Wanglong park
Yantaishan park
Cangshan education park
Hexie park
Sanjiangkou ecological park
South riverside park
Yafeng park
Jin 'an Jiangbin park
Party building theme park
Liuhuaxi park
Taiyuhe park
Jinniushan sports park
Minshui park
Fudao park
Fuzhou children's park
Yushan scenic park
Rongcheng square park
Zuohai park
Wenquan park
Xihu park
Yeshan park
Jiantianhu park
Xichan temple park
Chating park
Guangminggang park
Binfen park
Cangqian park
Taijiang jiangbin park
Shuishang park
Gaogaishan park
Douding sponge park
Guangminggang south park
Fukshan country park
Linpu park
Shita park
Aixiang park
Qiulong park
Wuyi square park
Jingdianhu park
Wenlinshan park
Aotan park
Yijing park
Wufeng park
Hubian park
Niugangshan park
Jinshan park
Xihe park
Nan park
Datiegang park
Fuzhou panda world park
Jiangxin park
Fenglingmu park
Wuyi sports park
South station square park

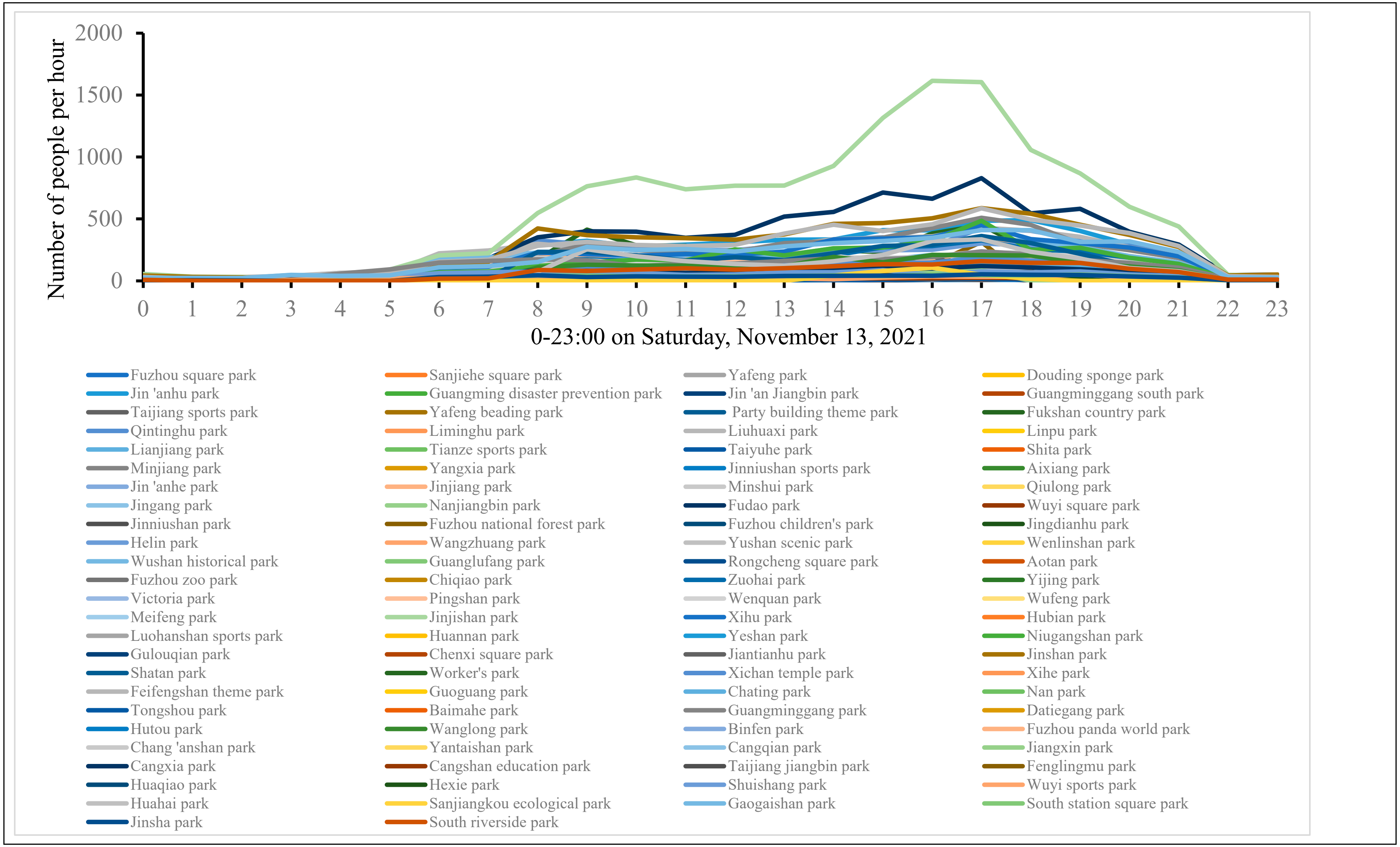

Number of people per hour
2000
1500
1000
500
0
0 1 2 3 4 5 6 7 8 9 10 11 12 13 14 15 16 17 18 19 20 21 22 23
0-23:00 on Saturday, November 13, 2021
Fuzhou square park
Sanjiehe square park
Yafeng park
Douding sponge park
Jin 'anhu park
Guangming disaster prevention park
Jin 'an Jiangbin park
Guangminggang south park
Taijiang sports park
Yafeng beading park
Party building theme park
Fukshan country park
Qintinghu park
Liminghu park
Liuhuaxi park
Linpu park
Lianjiang park
Tianze sports park
Taiyuhe park
Shita park
Minjiang park
Yangxia park
Jinniushan sports park
Aixiang park
Jin 'anhe park
Jinjiang park
Minshui park
Qiulong park
Jingang park
Nanjiangbin park
Fudao park
Wuyi square park
Jinniushan park
Fuzhou national forest park
Fuzhou children's park
Jingdianhu park
Helin park
Wangzhuang park
Yushan scenic park
Wenlinshan park
Wushan historical park
Guanglufang park
Rongcheng square park
Aotan park
Fuzhou zoo park
Chiqiao park
Zuohai park
Yijing park
Victoria park
Pingshan park
Wenquan park
Wufeng park
Meifeng park
Jinjishan park
Xihu park
Hubian park
Luohanshan sports park
Huannan park
Yeshan park
Niugangshan park
Gulouqian park
Chenxi square park
Jiantianhu park
Jinshan park
Shatan park
Worker's park
Xichan temple park
Xihe park
Feifengshan theme park
Guoguang park
Chating park
Nan park
Tongshou park
Baimahe park
Guangminggang park
Datiegang park
Hutou park
Wanglong park
Binfen park
Fuzhou panda world park
Chang 'anshan park
Yantaishan park
Cangqian park
Jiangxin park
Cangxia park
Cangshan education park
Taijiang jiangbin park
Fenglingmu park
Huaqiao park
Hexie park
Shuishang park
Wuyi sports park
Huahai park
Sanjiangkou ecological park
Gaogaishan park
South station square park
Jinsha park
South riverside park

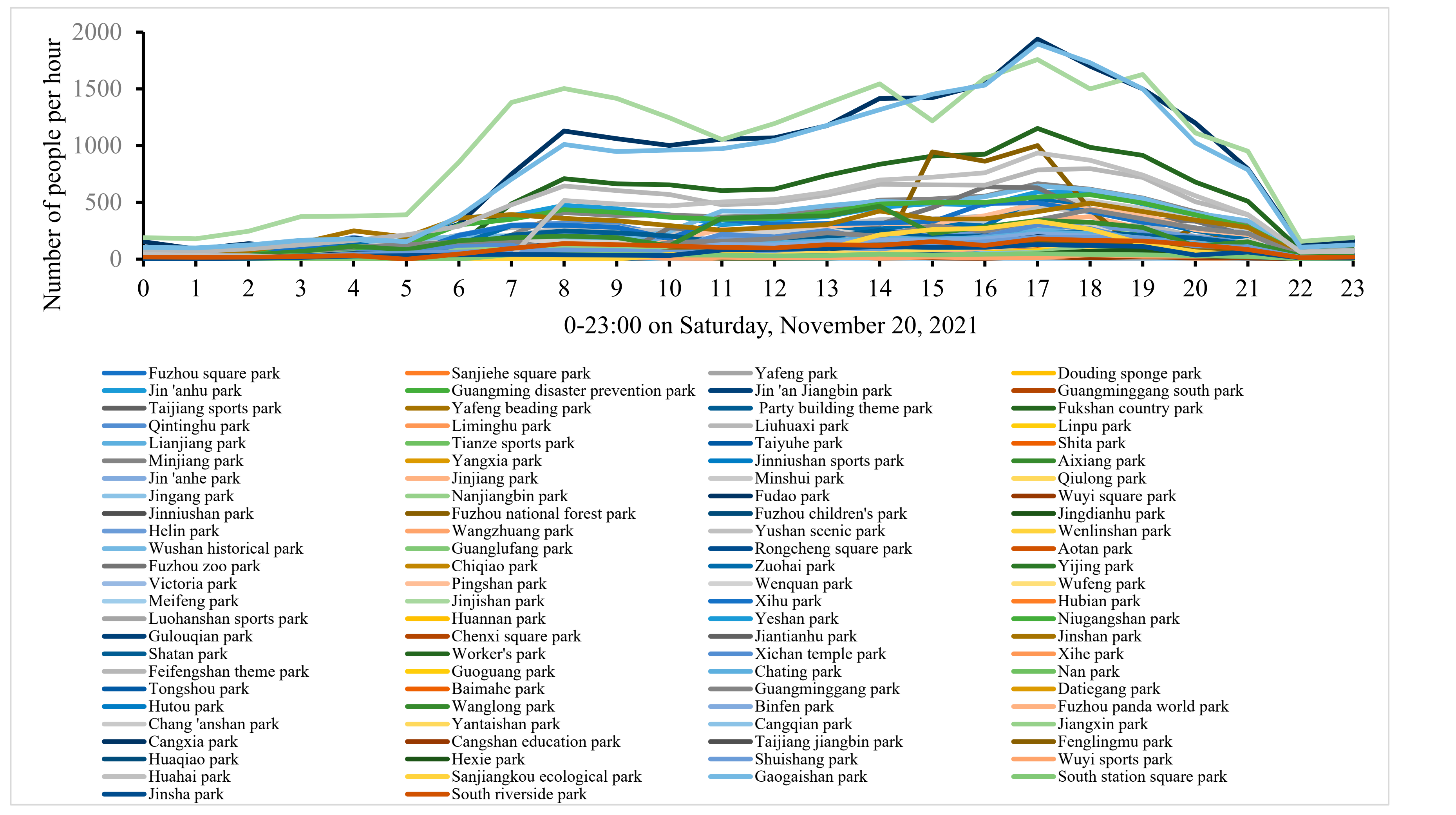

Figure S2 Hourly RTUD data on three consecutive Saturdays of parks in the study area

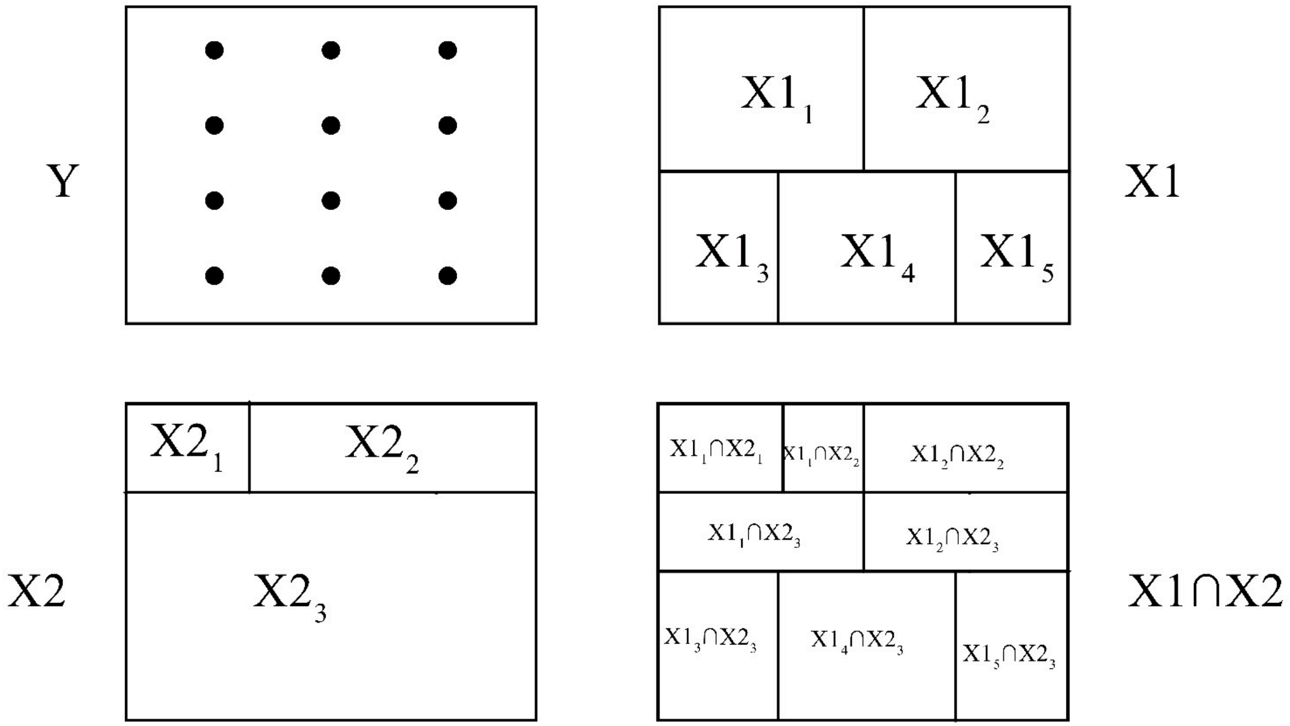

Figure S3 Interaction detection. Note: $q(X)$ and $q(X_1)$ are calculated respectively. Overlay the two layers X_1 and X_2 to get the new layer $X_1 n X_2$, calculate $q(X_1 \cap Y_2)$. Judge the interaction type of the two factors according to Table S2
